# miR-146a suppresses 5-lipoxygenase activating protein (FLAP) expression and Leukotriene B4 production in lung cancer cells

**DOI:** 10.18632/oncotarget.25482

**Published:** 2018-06-01

**Authors:** Joseph R. Iacona, Nicholas J. Monteleone, Carol S. Lutz

**Affiliations:** ^1^ Department of Microbiology, Biochemistry and Molecular Genetics, Rutgers Biomedical and Health Sciences, New Jersey Medical School and the School of Graduate Studies, Health Sciences Campus, Newark, NJ, USA

**Keywords:** lipoxygenase pathway, arachidonic acid, microRNA, gene expression, promoter methylation

## Abstract

Arachidonic acid (AA) can be converted into prostaglandins (PGs) or leukotrienes (LTs) by the enzymatic actions of cyclooxygenases (COX-1 and COX-2) or 5-lipoxygenase (5-LO), respectively. PGs and LTs are lipid signaling molecules that have been implicated in various diseases, including multiple cancers. 5-LO and its activating protein (FLAP) work together in the first two conversion steps of LT production. Previous work has suggested a role for LTs in cancer development and progression. MicroRNAs (miRNAs) are small RNA molecules that negatively regulate gene expression post-transcriptionally, and have previously been shown to be involved in cancer. Here, we show that high FLAP expression is associated with lower overall survival in lung adenocarcinoma patients, and FLAP protein is overexpressed in lung cancer cells compared to normal lung cells. Our lab has previously shown that miR-146a regulates COX-2 in lung cancer cells, and this miRNA is also predicted to target FLAP. Transient and stable transfections of miR-146a repress endogenous FLAP expression in lung cancer cells, and reporter assays show this regulation occurs through a direct interaction between the FLAP 3′ untranslated region (UTR) and miR-146a. Restoration of miR-146a also results in decreased cancer cell Leukotriene B4 (LTB_4_) production. Additionally, methylation analysis indicates the miR-146a promoter is hypermethylated in lung cancer cell lines. Taken together, this study and previous work from our lab suggest miR-146a is an endogenous dual inhibitor of AA metabolism in lung cancer cells by regulating both PG and LT production through direct targeting of the COX-2 and FLAP 3’ UTRs.

## INTRODUCTION

Arachidonic acid (AA) metabolism and subsequent eicosanoid production is an important biochemical pathway that is more complex than is apparent at first glance. Eicosanoids are 20-carbon lipid molecules produced as a result of the enzymatic activities of cyclooxygenases (COX-1 and COX-2) and 5-lipoxygenase (5-LO/ALOX5). There are two main arms of the metabolic pathway: AA is converted by the actions of COX-1 and COX-2 into prostaglandins (PGs) and thromboxanes (TXs) in one arm and the actions of 5-LO and its associated protein, 5-Lipoxygenase Activating Protein (FLAP/ALOX5AP), create leukotrienes (LTs) in the other arm of the pathway [1–3]. FLAP is localized at the nuclear membrane, and is necessary for the cellular production of LTs by binding to AA and presenting it to 5-LO, which then converts AA into an intermediate that is later catalyzed into bioactive LTs [1, 3]. AA metabolic enzymes are highly conserved in their amino acid sequence throughout mammalian evolution [4–6]. PGs, TXs, and LTs play roles in many homeostatic functions throughout the body, but also have pathophysiologic roles in cancer, inflammation, wound healing, asthma, allergic response, and bone fracture healing [7–15]. Thus, there is a need for meticulous regulation of these molecules.

COX-1 and COX-2 perform the same two enzymatic activities: oxidative cyclization and peroxidation of AA (2 and references therein). Our lab and others have demonstrated that COX-2 expression is regulated both transcriptionally and post-transcriptionally, including regulation by alternative polyadenylation of the COX-2 3′ untranslated region (3′ UTR) [16, 17] and microRNA (miRNA)-mediated regulation by several different miRNAs in multiple cell types and tissues [2, 18, 19]. Most notable for the purposes of the current study is the miR-146a-mediated suppression of COX-2 in lung cancer cells, as we have previously shown [19]. This regulation occurs through a direct and specific interaction between miR-146a and the COX-2 3′ UTR.

FLAP works in concert with 5-LO in two consecutive reactions to convert AA to the intermediate Leukotriene A4 (LTA_4_), which can be processed by a number of other enzymes into various LTs. LTs have long been studied in asthma and allergies, but recent evidence has implicated a role for them in cancer. Production of Leukotriene B4 (LTB_4_), one of the most potent leukotrienes, is typically restricted to leukocytes, yet is synthesized in various diseased epithelial cells, including lung cancer cells [20–23]. LTB_4_ can stimulate cell proliferation and promote cell survival in both colon and pancreatic cancer cell lines, and in general supports a favorable microenvironment for tumor growth and metastasis [13, 24–26].

Although more is known about 5-LO gene expression mechanisms, regulation of FLAP is no less important. Aberrant regulation of these molecules has been implicated in cancer development and progression [13, 27]. The pro-tumorigenic nature of LT metabolism has been demonstrated in numerous cancers, including but not limited to colorectal, lung, pancreatic, prostate, and chronic myeloid leukemia [28–32]. In addition, high FLAP expression in human breast cancer samples was correlated with decreased survival, and inhibiting FLAP activity attenuated breast tumor cell growth [33, 34]. Previous work has shown transcriptional activation to be key for regulation of FLAP [35–38], however, the role of post-transcriptional regulation has only recently been appreciated. The FLAP mRNA 3’ UTR contains several computationally predicted miRNA binding sites. miR-335 and miR-199a-5p were described as direct regulators of FLAP expression in human brain endothelial cells [39]. Interestingly, the FLAP 3′ UTR contains a predicted, highly conserved miR-146a binding site. We postulated that perhaps miR-146a might also regulate the other arm of the AA metabolic pathway, that is, the LT side in addition to the PG side. A single miRNA regulating both arms of a basic biochemical pathway would be an elegant and novel discovery.

Here, we demonstrate for the first time that FLAP is overexpressed in lung cancer. Cancer database analysis suggests a correlation between FLAP expression and overall survival in lung adenocarcinoma patients. This work also identifies FLAP as a novel target of miR-146a, as this miRNA can downregulate FLAP expression through binding its 3’ UTR. miR-146a treatment can also cause a significant decrease in LTB_4_ production. Finally, we show hypermethylation of the miR-146a promoter is responsible for its reduced expression in lung cancer cells. Taken together, these results highlight the discovery of miR-146a-mediated regulation of both arms of the AA metabolic pathway.

## RESULTS

### High FLAP expression is negatively correlated with overall survival in lung adenocarcinoma patients

In order to investigate the effect of FLAP expression in lung cancer patients, we utilized the Kaplan-Meier Plotter online tool (www.kmplot.com), a survival analysis software designed to identify cancer biomarkers with prognostic value based on patient transcriptomic data compiled from multiple studies. We consulted the version specific for non-small cell lung cancer (NSCLC) [40]. FLAP (ALOX5AP) gene expression was analyzed, and patients were categorized as having low or high expression based on how their FLAP level related to the median value.

**Figure 1:**
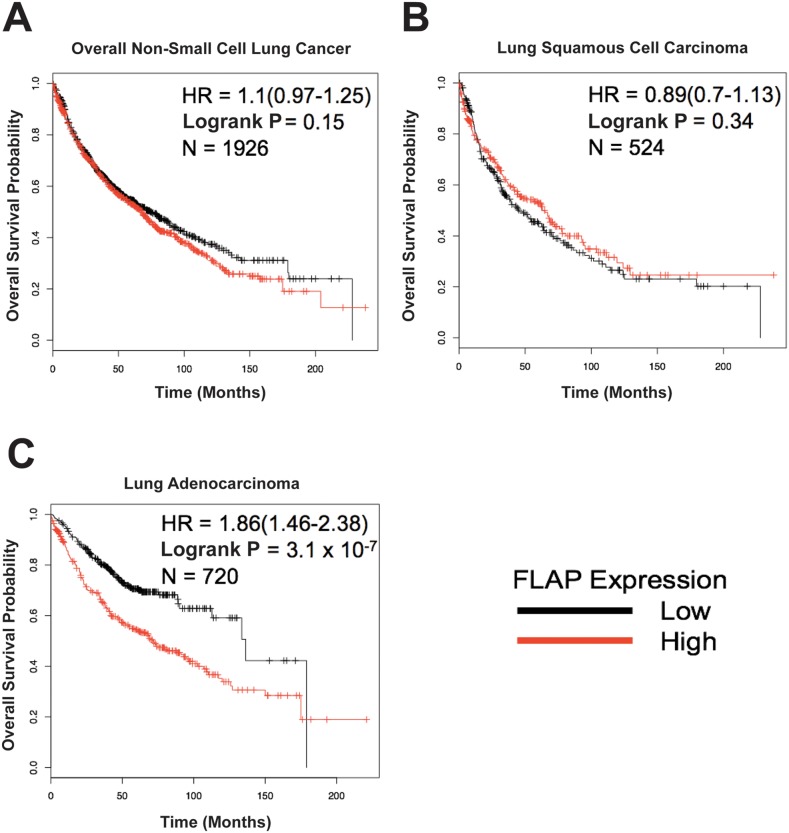
5-Lipoxygenase Activating Protein (FLAP) expression may have prognostic value in lung adenocarcinoma The Non-Small Cell Lung Cancer (NSCLC) KM Plotter Tool (http://www.kmplot.com) was used to generate survival curves based on a patient's overall survival in months and their FLAP expression level (low or high) relative to the median value. **(A)** No significant correlation between FLAP expression and overall survival in 1,926 NSCLC patients (*P* = 0.15). **(B)** No significant correlation between FLAP expression and overall survival in 524 lung squamous cell carcinoma patients (*P* = 0.34). **(C)** Highly significant correlation between FLAP expression and overall survival in 720 lung adenocarcinoma patients (*P* = 3.1 × 10^-7^).

Analysis of data from 1,926 NSCLC patients showed a trend of high FLAP expression correlating with a lower overall probability of survival, but this was not statistically significant (*P* = 0.15, HR = 1.1) (Figure 1A). 1,244 of these patients had an NSCLC subtype associated with their data. Upon subtype-specific analysis no significant difference in overall survival was seen in the 524 patients with lung squamous cell carcinoma (*P* = 0.34, HR = 0.89) (Figure 1B). However, there was a highly significant correlation between high FLAP expression and lower overall probability of survival in the 720 patients with lung adenocarcinoma (*P* = 3.1 × 10^-7^, HR = 1.86) (Figure 1C). These data preliminarily suggest FLAP expression may be used as a prognostic biomarker in lung adenocarcinoma. Why these results are subtype-specific is unclear, and is an interesting point for future investigation.

### FLAP expression in lung cell lines

**Figure 2:**
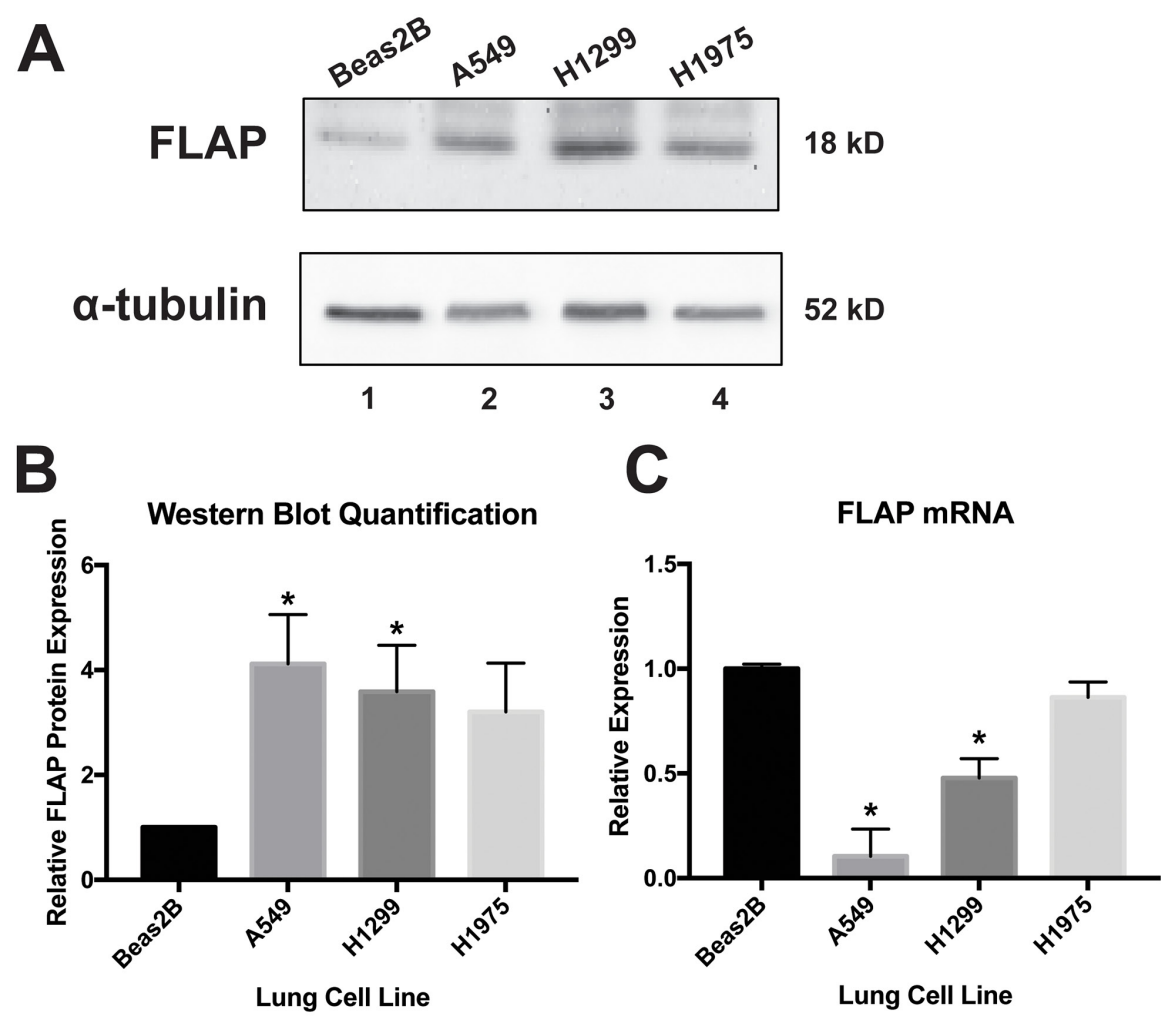
FLAP expression levels in lung cell lines **(A)** Western blot analysis of Beas2B (normal lung), A549 (NSCLC), H1299 (NSCLC), and H1975 (NSCLC) cell lysates. Western blots were repeated at least three times. **(B)** Quantification of relative FLAP protein levels was performed using the gel analysis tool on ImageJ software and normalized to α-tubulin protein levels. Analysis indicated significant overexpression of FLAP protein in A549 (*lane 2*) and H1299 cells (*lane 3*), but not H1975 cells (*lane 4*) with respect to Beas2B cells (*lane 1*). (^*^) *P* < 0.04, *n*=3. **(C)** ΔΔCT qRT-PCR analysis indicated significantly increased expression of FLAP mRNA in Beas2B cells compared to A549 and H1299 cells, but not H1975 cells. FLAP expression was normalized to GAPDH mRNA. (^*^) *P* < 0.01, *n*=3.

Our laboratory and others have reported COX-2 overexpression in various cancer cells (19 and references therein). Increased 5-LO expression also has been demonstrated in various cancers [28–32]. However, the pro-cancer role of FLAP has only been focused on in detail in the context of breast cancer [33, 34]. In order to establish FLAP protein levels in lung cell lines, Western blot analysis was performed on lysates from A549, H1299, and H1975 cells (lung adenocarcinoma) and compared to lysates from Beas2B cells (normal immortalized lung). As seen in Figure 2A, 2B, FLAP protein is significantly upregulated in A549 and H1299 cells compared to Beas2B cells, suggesting a potential role for FLAP in lung adenocarcinoma. FLAP protein is also upregulated in H1975 cells, but the data were not statistically significant.

In order to establish the relative abundance of FLAP mRNA in these lung cells, real-time quantitative reverse transcriptase PCR (qRT-PCR) was performed. Comparative Cycle Threshold (CT) (ΔΔCT) data analysis revealed that Beas2B cells express significantly more FLAP mRNA than A549 and H1299 cells, but no significant difference was observed when compared to H1975 cells (Figure 2C). These findings are intriguing, since they show an inverse expression pattern between FLAP mRNA and protein (Figure 2B). Maier et al. analyzed the literature and determined that, in general, an overall poor correlation exists between mRNA and protein levels in various cell types and species. The authors propose multiple reasons for this: 1) post-transcriptional regulation, 2) post-translational regulation, and 3) background noise and experimental error [41]. Our data suggest post-transcriptional and/or translational regulation is occurring in the normal lung cells, leading to a lower relative level of FLAP protein despite a higher abundance of its mRNA. This regulation may be reduced or lost in the lung cancer cells, resulting in overexpression of FLAP protein.

### FLAP is a predicted target of miR-146a

**Figure 3:**
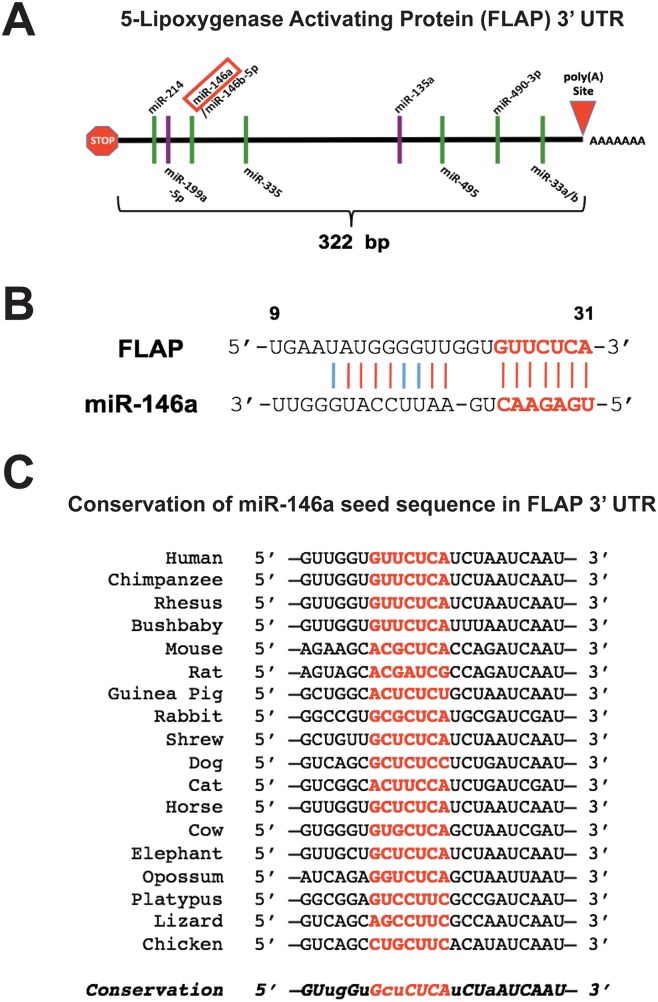
miR-146a is predicted to target the FLAP 3′ UTR **(A)** Schematic illustration of the FLAP 3′ UTR (not drawn to scale). The stop sign and red triangle represent the stop codon and polyadenylation site, respectively. Green lines indicate putative miRNA binding sites as predicted by the microRNA.org and TargetScan algorithms. Purple lines indicate miRNA binding sites validated in the literature. **(B)** Predicted alignment of miR-146a binding to the FLAP 3′ UTR. The 7-mer seed sequence is highlighted in red and bold. Red lines indicate perfect complementarity and light blue lines indicate low-affinity U-G pairing. Numbers represent the base position within the 3′ UTR. **(C)** Diagram displaying the sequence conservation of the miR-146a seed sequence (highlighted in red) in the FLAP 3′ UTR across various vertebrate species. Uppercase italic letters indicate conservation over at least 12 vertebrate species; lowercase italic letters indicate conservation over at least 9 vertebrate species. Sequences were obtained from TargetScan.

Multiple forms of post-transcriptional regulation may be at play resulting in this disparity between FLAP mRNA and protein expression. There are two predicted splice variants of FLAP, both of which have the same 322 bp 3′ UTR (Figure 3A and [2]). Expression of the protein isoform of the second splice variant has yet to be experimentally validated. The FLAP 3′ UTR contains several putative miRNA binding sites. The miRNAs depicted in Figure 3A (binding sites represented as green lines) are predicted to target the FLAP 3′ UTR based on two computational algorithms: miRanda (www.microrna.org) and TargetScan (www.targetscan.org). One such miRNA is miR-146a. This miRNA was of particular interest to us because we previously demonstrated that miR-146a directly regulates COX-2 expression and prostaglandin production in these lung cells [19]. The abovementioned algorithms suggest miR-146a can also directly regulate FLAP, another protein involved in inflammation and AA metabolism. Figure 3B shows the predicted alignment between mature miR-146a and the FLAP 3′ UTR. The miR-146a-FLAP mRNA duplex has high complementarity at both the seed region (5′ end of the mature miRNA) and the miRNA 3′ end. This implies a high likelihood that miR-146a can bind to FLAP mRNA [42]. This potential interaction is further supported by the high evolutionary conservation of the miR-146a 7-mer seed sequence in the 3′ UTR of FLAP orthologs in various vertebrate species (Figure 3C).

**Figure 4:**
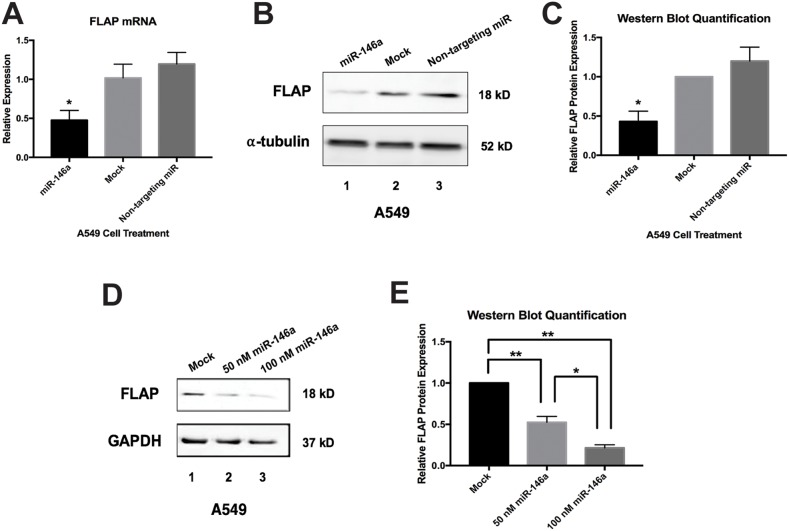
Synthetic miR-146a represses endogenous FLAP mRNA and protein in A549 cells A549 cells were transiently transfected with miRNA mimics. Cells were lysed and RNA and protein were isolated 48 hours post-transfection. **(A)** ΔΔCT qRT-PCR analysis indicated decreased FLAP mRNA expression in A549 cells transfected with 50 nM miR-146a. FLAP expression was normalized to GAPDH mRNA. FLAP mRNA levels were significantly different in cells transfected with miR-146a from levels in mock-treated cells and cells transfected with 50 nM non-targeting miRNA. (^*^) *P* < 0.01, *n*=3. **(B)** Western blot analysis of cell lysates indicated decreased FLAP protein expression in A549 cells transfected with 50 nM miR-146a (*lane 1*) compared to mock-treated A549 cells (*lane 2*) and A549 cells transfected with 50 nM non-targeting miRNA (*lane 3*). A representative blot is shown of three independent experiments. **(C)** Quantification of relative FLAP protein levels was performed using the gel analysis tool on ImageJ software and normalized to α-tubulin protein levels. (^*^) *P* < 0.01, *n*=3. **(D)** Western blot analysis of cell lysates indicated dose-dependent decreased FLAP protein expression in A549 cells transfected with 50 and 100 nM miR-146a (*lanes 2 and 3*) compared to mock-treated A549 cells (*lane 1*). A representative blot is shown of three independent experiments. **(E)** Quantification of relative FLAP protein levels was performed using the gel analysis tool on ImageJ software and normalized to GAPDH protein levels. (^*^) *P* < 0.035, (^**^) *P* < 0.025, *n*=3.

miR-146a expression is significantly downregulated in A549, H1299, and H1975 cells compared to Beas2B cells (see Figure 4 of reference [19]). Our lab previously established an inverse relationship between miR-146a and COX-2 protein expression in these lung cells [19]. A similar inverse relationship exists between miR-146a and FLAP protein expression. Thus, we hypothesize that miR-146a regulates FLAP mRNA and therefore is involved in the resulting expression of FLAP protein. It is important to note that in our cells of interest, no mutation is present in the miR-146a binding site in the FLAP or COX-2 3′ UTRs (data not shown) that may be responsible for the dysregulation seen in the cancer cells.

### Introduction of synthetic miR-146a represses endogenous FLAP expression

To determine the effect of exogenous miR-146a on endogenous FLAP mRNA and protein, A549 cells were transfected with 50 nM synthetic mature miR-146a. Another subset of A549 cells was transfected with 50 nM of a commercially available miRNA mimic with a sequence predicted to not target the human transcriptome. This non-targeting miRNA served as a control for non-specific repression. All samples were compared to a mock-transfected group of A549 cells treated with transfection reagent alone. Protein and RNA were isolated from the cells 48 hours post-transfection. qRT-PCR was performed for FLAP mRNA expression and ΔΔCT data analysis revealed a significant decrease in FLAP mRNA expression in A549 cells transfected with miR-146a (Figure 4A). Western blot analysis showed a similar repression of endogenous FLAP protein in miR-146a-treated cells (Figure 4B, 4C). The non-targeting miR showed no significant effect on FLAP mRNA or protein expression (Figure 4). In addition, increasing the miR-146a mimic concentration to 100 nM showed a dose-dependent effect on FLAP protein levels (Figure 4D, 4E). Since miR-146a transfection resulted in a decrease in both FLAP mRNA and protein levels, our data do not conclusively prove whether miR-146a is mechanistically downregulating FLAP expression via mRNA degradation, translational repression, or both. However, it is clear that endogenous FLAP can be downregulated by miR-146a in A549 lung cells.

### Inducible miR-146a expression also decreases FLAP protein levels

**Figure 5:**
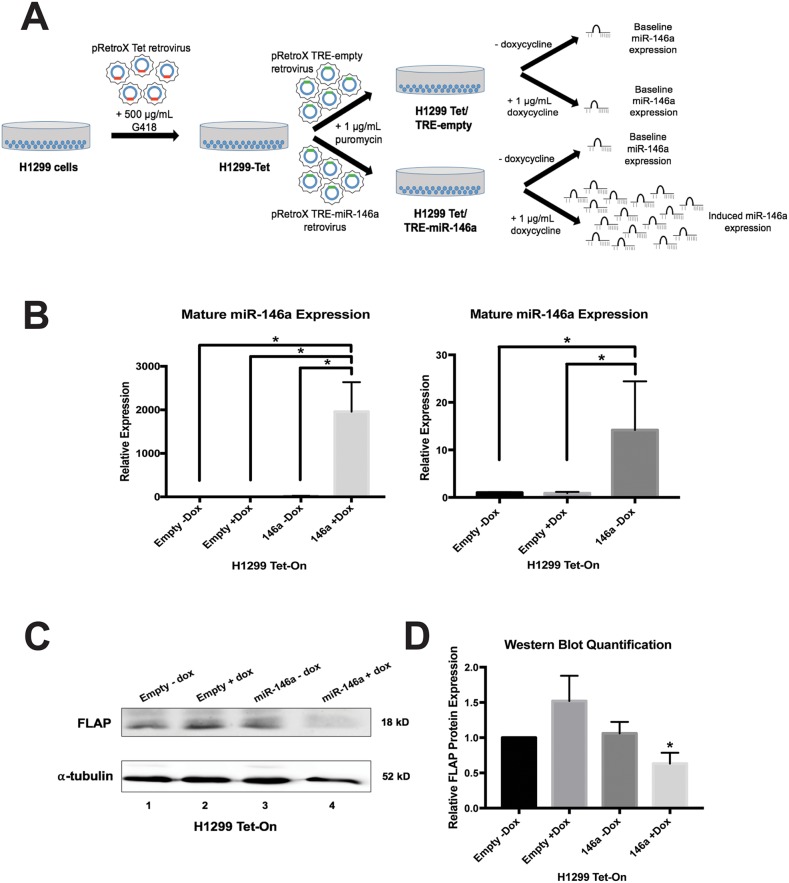
Doxycycline-induced expression of miR-146a in H1299 cells represses endogenous FLAP protein **(A)** Schematic illustration of methodology used for stable cell line development (see methods section for more details). Where indicated, H1299 Tet/TRE-empty and H1299 Tet/TRE-miR-146a cells were cultured in 1 μg/mL doxycycline. **(B)** *Left:* ΔΔCT qRT-PCR analysis indicated successful induction of mature miR-146a expression in H1299 Tet/TRE-miR-146a cells. miR-146a expression was normalized to U6 snRNA expression. *Right:* Focused graph showing miR-146a expression in control cell lines. (^*^) *P* < 0.03, *n*=3. **(C)** Western blot analysis of cell lysates indicated decreased FLAP protein expression only in H1299 Tet/TRE-miR-146a cells cultured with doxycycline. A representative blot is shown of three independent experiments. **(D)** Quantification of relative FLAP protein levels was performed using the gel analysis tool on ImageJ software and normalized to α-tubulin protein levels. (^*^) *P* < 0.037, *n*=3.

miRNA mimic experiments are limited by their transient nature. To examine the long-term effects of miR-146a restoration on FLAP expression, stable clones of H1299 cells were generated with miR-146a expression under the control of the Tet Response Element (TRE). Using the Tet-On system, miR-146a overexpression occurs only when the cells are cultured in the presence of doxycycline (Figure 5A). Like A549 cells, H1299 cells are also derived from lung adenocarcinoma.

Using retroviral transduction, H1299 cells were generated expressing either the TRE empty vector or the TRE-miR-146a expression cassette (Figure 5A, Supplementary Figure 1). No significant difference in mature miR-146a expression was observed between H1299 TRE-empty cells cultured with or without 1 μg/mL doxycycline (Figure 5B, right), controlling for the effect of this concentration of doxycycline. Relative to H1299 TRE-empty cells, mature miR-146a expression robustly increased (~2000-fold) in the H1299 TRE-miR-146a cells cultured in doxycycline (Figure 5B, left). Slight “leaky expression” was apparent, as H1299 TRE-miR-146a cells cultured without doxycycline showed a ~14-fold increase in mature miR-146a expression (Figure 5B, right). Despite this leaky expression, a significant decrease in endogenous FLAP protein levels was only seen in H1299 TRE-miR-146a cells cultured in the presence of doxycycline (Figure 5C, 5D), suggesting higher degrees of induction are needed to see an effect on protein levels. This was similar to the observed repression in A549 cells transiently transfected with synthetic miR-146a (Figure 4). Using both a different cell line and expression method, these results support a role for miR-146a in the negative regulation of FLAP expression in lung cancer cell lines.

### Reporter protein activity is affected by direct miR-146a interaction with the FLAP 3′ UTR

**Figure 6:**
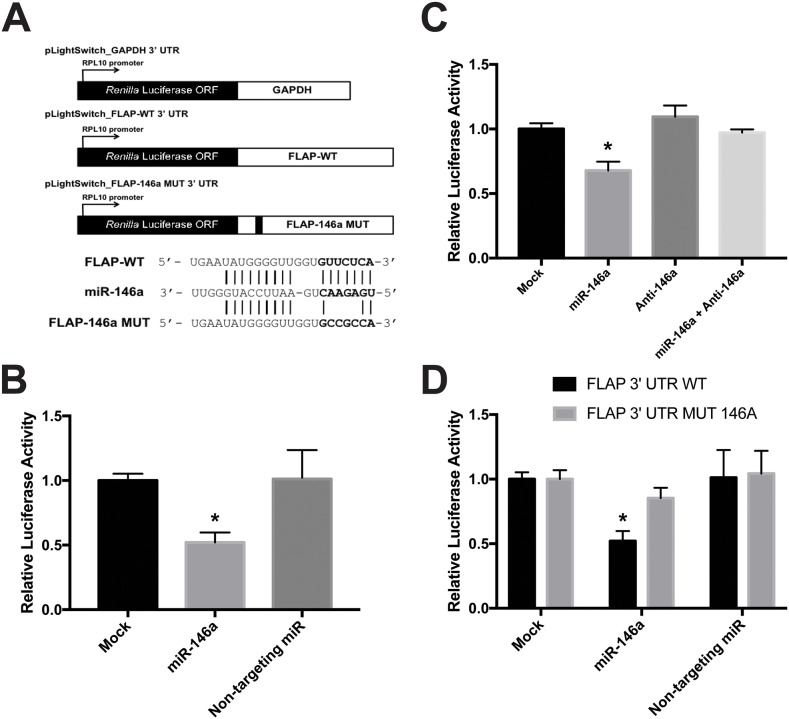
miR-146a directly targets the FLAP 3′ UTR (**A**, *top*) Schematic illustration of the *Renilla* luciferase 3′ UTR constructs used: pLightSwitch_GAPDH 3′ UTR, pLightSwitch_FLAP-WT 3′ UTR, and pLightSwitch_FLAP-146a MUT 3′ UTR. (A, *bottom*) Predicted alignment of miR-146a binding to the FLAP WT 3′ UTR. The 7-mer seed sequence is highlighted in bold. Lines indicate perfect complementarity and potential low-affinity U-G pairing. The alignment is also shown for miR-146a binding to the FLAP 3′ UTR containing a 4-nt mutation (UUCU-CCGC) in the FLAP-146a MUT 3′ UTR construct. **(B)** *Renilla* luciferase activity was measured in HeLa cells transfected with 50 nM synthetic miRNAs (miR-146a or non-targeting miR) and the pLightSwitch_FLAP-WT 3′ UTR construct. FLAP WT 3′ UTR luciferase activity was normalized to GAPDH 3′ UTR luciferase activity in HeLa cells exposed to the same miRNA condition. Luciferase activity was further normalized to total protein concentration of each sample. *Renilla* luciferase activity was significantly decreased only in samples treated with miR-146a. (^*^) *P* < 0.002, *n*=6. **(C)** FLAP-WT 3′ UTR *Renilla* luciferase activity was measured in HeLa cells transfected with 50 nM miR-146a alone, 50 nM anti-miR-146a alone, or 50 nM of both miR-146a and anti-miR-146a. Luciferase activity was normalized as described above in *B*. *Renilla* luciferase activity in cells treated with miR-146a and its antagomiR together was significantly greater than in cells treated with miR-146a alone. (^*^) *P* < 0.001, *n*=5. **(D)** FLAP-WT 3′ UTR and FLAP-146a MUT 3′ UTR *Renilla* luciferase activities were measured in HeLa cells transfected with 50 nM synthetic miRNAs (miR-146a or non-targeting miR). Luciferase activity was normalized as described above in *B*. FLAP-146a MUT 3′ UTR *Renilla* luciferase activity was significantly higher than FLAP-WT 3′ UTR *Renilla* luciferase activity in cells treated with miR-146a due to the mutation in the miR-146a binding site. (^*^) *P* < 0.01, *n*=3.

To examine whether the effect of miR-146a on FLAP expression is directly controlled through its 3′ UTR, luciferase reporter assays were performed. We purchased the pLightSwitch_3UTR *Renilla* luciferase reporter construct from Switch Gear Genomics. This plasmid contains the *Renilla* luciferase open reading frame (ORF) under the control of the constitutively active RPL10 promoter. We cloned the full-length FLAP 3′ UTR (pLightSwitch_FLAP-WT 3′ UTR) and GAPDH 3′ UTR (pLightSwitch_GAPDH 3′ UTR) downstream of the *Renilla* luciferase ORF (Figure 6A). These reporter assays were carried out in HeLa cells to avoid interference from endogenous FLAP mRNA levels in Beas2B, A549, or H1299 cells. HeLa cells do not express FLAP mRNA or protein (data not shown). The cells were mock-treated or transfected with synthetic miR-146a or non-targeting miR. Transfection with the abovementioned luciferase constructs was performed afterwards. Relative FLAP 3′ UTR *Renilla* luciferase activity decreased ~50% only in the cells treated with miR-146a (Figure 6B). These data suggest that the observed repression of FLAP expression (Figure 4 and 5) is carried out by miR-146a via the FLAP 3′ UTR.

Next, the FLAP 3′ UTR *Renilla* luciferase assay was repeated in the presence of a miR-146a antagomiR (anti-miR-146a) in order to ensure our observations were specific. This antagomiR works by sequestering miR-146a and preventing it from interacting with its targets. HeLa cells were mock-treated or transfected with miR-146a alone, anti-miR-146a alone, or miR-146a/anti-miR-146a together. Luciferase construct transfection followed, as above. When the antagomiR sequestered miR-146a, the relative FLAP 3′ UTR *Renilla* luciferase activity significantly increased to similar levels as the mock-treated cells. In addition, anti-miR-146a alone did not affect luciferase activity (Figure 6C). These results further support a specific interaction between miR-146a and the FLAP 3′ UTR that consequentially drives FLAP downregulation.

In order to further understand the nature of the miR-146a effect, it was necessary to determine whether miR-146a directly interacts with the FLAP 3′ UTR through a specific sequence. The FLAP 3′ UTR contains a single computationally predicted miR-146a binding site that begins ~10-15 nucleotides downstream of the stop codon (Figure 3). Literature suggests the seed region is crucial for miRNA function [42]. Therefore, 4 nucleotides in the 7-mer miR-146a seed sequence in the FLAP 3′ UTR luciferase construct were mutated using site-directed mutagenesis (TTCT to CCGC) (Figure 6A). HeLa cells were transfected with synthetic miRs and luciferase constructs (pLightSwitch_FLAP-146a MUT) as described previously. As seen in Figure 6D, miR-146a was able to downregulate relative luciferase activity of the FLAP-WT 3′ UTR construct as shown previously (Figure 6B). However, relative luciferase activity was significantly higher in cells transfected with miR-146a plus the FLAP 3′ UTR construct containing a mutated miR-146a seed sequence (Figure 6D). These observations suggest miR-146a-mediated repression of FLAP expression is executed through a direct interaction between miR-146a and a binding site in the FLAP 3′ UTR.

### Leukotriene B4 production is decreased by introduction of miR-146a

**Figure 7:**
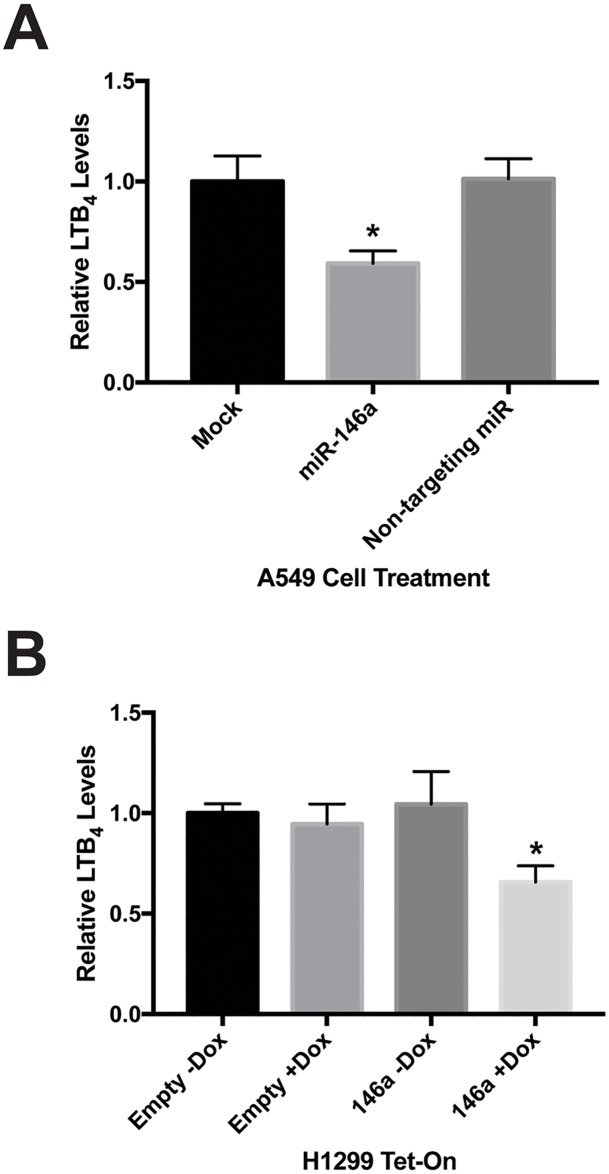
Increased miR-146a levels result in decreased cellular production of LTB_4_ **(A)** A549 cells were transiently transfected with 50 nM miRNA mimics. Cell-free, serum-free supernatants were collected 48 hours post-transfection. An enzyme-linked immunosorbent assay (ELISA) was used to measure Leukotriene B4 (LTB_4_) concentrations. A549 cells transfected with miR-146a produced significantly less LTB_4_ compared to mock-treated cells and cells transfected with a non-targeting miRNA. (^*^) *P* < 0.025, *n*=3. **(B)** Where indicated, H1299 Tet/TRE-empty and H1299 Tet/TRE-miR-146a cells were cultured in 1 μg/mL doxycycline. Cell-free, serum-free supernatants were collected and an ELISA was used to measure LTB_4_ concentrations. H1299 Tet/TRE-miR-146a cells cultured in doxycycline produced significantly less LTB_4_. (^*^) *P* < 0.04, *n*=3.

FLAP and 5-LO work in concert to convert AA to the intermediate LTA_4_, which can be processed by a number of other enzymes into various LTs. One of these lipid signaling molecules is LTB_4_. LTB_4_ has been described as a pro-inflammatory and pro-tumorigenic LT, as well as one of the most potent LTs [13]. We previously showed A549 cells produce lower levels of Prostaglandin E_2_ (PGE_2_) following miR-146a transfection [19]. Because our data showed miR-146a transfection resulted in modulated levels of FLAP protein, we were interested to see if this downregulation also affects LTB_4_ production. To investigate this, we performed an enzyme-linked immunosorbent assay (ELISA) specific for LTB_4_. This assay was carried out on mock-treated A549 cells and cells transiently transfected with miR-146a or a non-targeting miR. Analysis revealed a significant decrease in LTB_4_ release in cells treated with miR-146a. The non-targeting miR did not display this effect (Figure 7A). We also performed this assay on the H1299 Tet-On stable cells and observed significantly reduced LTB_4_ production only in the H1299 TRE-146a cells cultured in the presence of doxycycline (Figure 7B). These results imply miR-146a-mediated regulation of FLAP also causes a decrease in its biological function. It is important to note that in a previous study, our lab had shown transfection of synthetic miRNAs at a 50 nM concentration in A549 cells does not result in significant levels of cytotoxicity that could potentially account for the observed decrease in eicosanoid production (see Figure 7B of reference [19]).

### The miR-146a promoter is highly methylated at CpG sites in lung cancer cell lines

**Figure 8:**
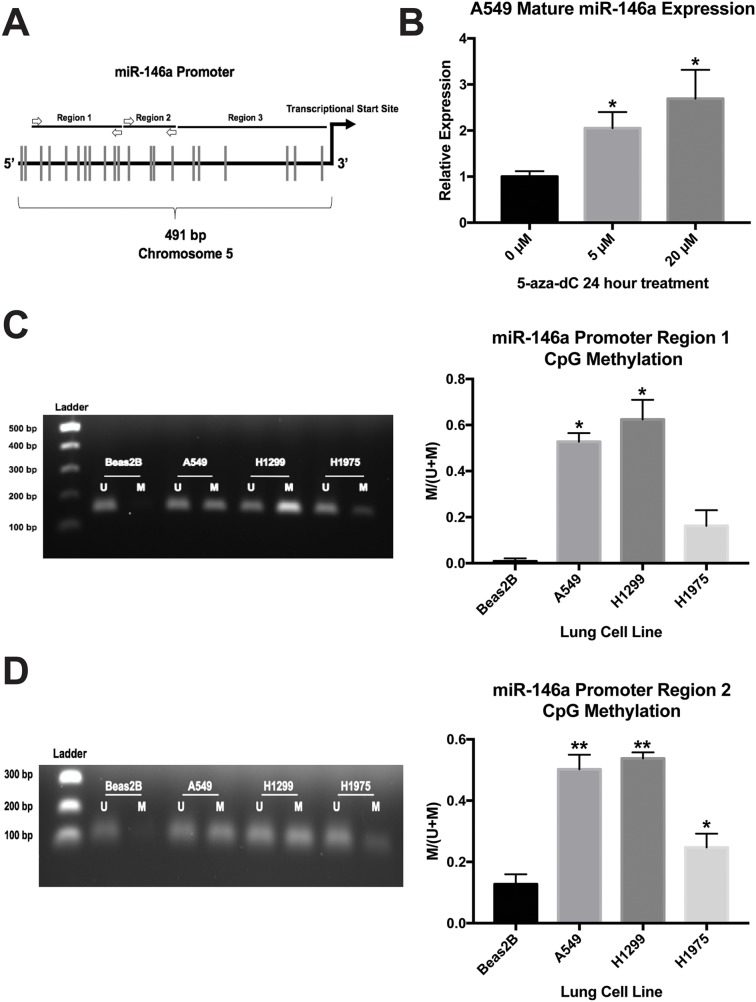
miR-146a expression is regulated by CpG methylation in lung cell lines **(A)** Schematic illustration depicting the miR-146a promoter region (not drawn to scale). Vertical lines indicate CpG sites. Primer annealing locations for methylation-specific PCR (MSP) are shown as white arrows. **(B)** ΔΔCT qRT-PCR analysis indicated increased expression of mature miR-146a in A549 cells following 5-aza-2’-deoxycytidine (5-aza-dC) treatment. miR-146a expression was normalized to U6 snRNA expression. (^*^) *P* < 0.03, *n*=3. **(C)** *Left:* MSP products for miR-146a promoter region 1 in four lung cell lines run on a 1% agarose gel. Representative gel of three independent experiments is shown. *Right:* Image J software was used to measure band intensities. The intensity of the methylated band divided by total band intensity for each cell line is shown. (^*^) *P* < 0.006, *n*=3. **(D)** *Left:* MSP products for miR-146a promoter region 2 in four lung cell lines run on a 1% agarose gel. Representative gel of three independent experiments is shown. *Right:* Image J software was used to measure band intensities. The intensity of the methylated band divided by total band intensity for each cell line is shown. (^*^) *P* < 0.025, (^**^) *P* < 0.001, *n*=3. U: unmethylated state; M: methylated state.

We previously established that miR-146a expression is significantly downregulated in A549, H1299, and H1975 cells compared to Beas2B cells (see Figure 4 of reference 19), but the mechanism for this remained unclear. It is important to note that in our cells of interest, no mutation is present in the pri-miR-146a genomic sequence, nor is there a miRNA processing defect, that may be responsible for the dysregulation seen in the cancer cells (data not shown). miR-146a expression is suppressed in lung cancer cell lines despite being an NF-κB-dependent gene [43], a pathway which is often constitutively active in lung cancer [44]. Because hypermethylation of tumor suppressor gene promoters is frequently observed in cancers, we predicted that this may be responsible for lower miR-146a levels in lung cancer. We analyzed a 491 bp genomic sequence upstream of the miR-146a transcriptional start site which was identified by David Baltimore's lab as the miR-146a promoter [43]. The promoter sequence contains 21 CpG sites (Figure 8A).

Next, we treated A549 cells for 24 hours with 5-aza-2’-deoxycytidine, which inhibits DNA methyltransferase activity. qRT-PCR analysis indicated this treatment resulted in a 2-3 fold increase in mature miR-146a expression (Figure 8B), suggesting DNA methylation may be playing a role in miR-146a expression in A549 cells. In order to detect DNA methylation status, we designed methylation-specific PCR (MSP) primers for two regions of the miR-146a promoter sequence. These assays revealed that A549 and H1299 cells had significantly higher methylation levels at these two regions than Beas2B cells (Figure 8C, 8D). H1975 cells also had a higher methylation status, but the data were only statistically significant for region 2 (Figure 8C, 8D). Region 3 does not have a sufficient density of CpG sites for MSP analysis. Our results suggest promoter CpG hypermethylation is responsible for repressed miR-146a expression in lung cancer cell lines.

Overall, our data show miR-146a can downregulate FLAP expression in lung cancer cells by directly targeting its 3′ UTR. This modulation in FLAP protein levels results in decreased lung cancer cell production of LTB_4_, and miR-146a expression is regulated by DNA methylation.

## DISCUSSION

In this communication, we have identified FLAP as a novel target of miR-146a. We have demonstrated that an inverse relationship exists between FLAP protein expression and miR-146a expression in lung cancer cell lines as compared to Beas2B cells. In lung cancer cell lines, the FLAP expression level is high (Figure 2) but the miR-146a levels are low [19]. In contrast, Beas2B cells show robust expression of miR-146a but low FLAP protein expression (Reference [19] and Figure 2). In silico analyses suggest FLAP expression levels negatively correlate with lung adenocarcinoma patient overall survival (Figure 1) and the miR-146a binding site in the FLAP 3’ UTR is highly conserved throughout evolution (Figure 3). Transient (Figure 4) and stable (Figure 5) transfection of miR-146a in lung cell lines resulted in reduced endogenous FLAP expression. Luciferase assays demonstrated the direct and specific nature of the interaction of miR-146a with FLAP mRNA (Figure 6). The biological effect of miR-146a on FLAP was revealed by the observation of significantly reduced LTB_4_ production in the presence of miR-146a (Figure 7) as compared to control treatments. Finally, CpG methylation is the likely mechanism responsible for suppressed miR-146a expression in lung cancer cells (Figure 8).

**Figure 9:**
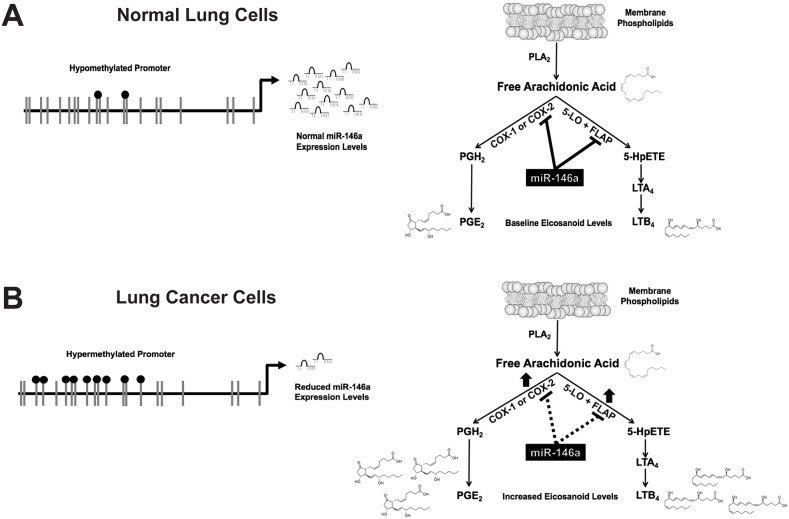
miR-146a negatively regulates both the prostaglandin and leukotriene arms of the arachidonic acid metabolic pathway **(A)** In normal lung cells, a hypomethylated promoter allows miR-146a to be expressed. miR-146a directly targets COX-2 and FLAP, which controls PGE_2_ and LTB_4_ production. **(B)** In lung cancer cells, a hypermethylated promoter results in reduced miR-146a expression. This leads to increased COX-2 and FLAP protein expression, and therefore higher levels of PGE_2_ and LTB_4_. Adapted in part from: [14].

Our data suggest a model, shown in Figure 9. Expression of miR-146a is regulated by promoter CpG methylation. Methylation is increased in lung cancer cells, resulting in significantly less mature miR-146a. This leads to increased protein levels of miR-146a target genes COX-2 [19] and FLAP (this work), which ultimately allows the cell to produce greater amounts of PGE_2_ and LTB_4_. Post-transcriptional regulation of COX-2 and FLAP via miR-146a is an endogenous mechanism by which the cell can control metabolism of AA into both PGs and LTs. The biological significance of targeting both arms of the pathway has recently become apparent in multiple reports discussing AA shunting. The concept of shunting was first suggested when researchers observed that inhibition of one arm of the pathway lead to upregulation of the other arm. One group noted that when they induced an acute inflammatory response in 5-LO knockout mice, administering a cyclooxygenase inhibitor was able to resolve the inflammation, but it was unable to do so in wild-type mice [45]. Guo et al. reported that COX-2 was upregulated in 5-LO knockout mice that had oral cancer induced with ethanol [46]. Manigrasso and O’Connor observed 4-fold higher levels of LTs in femur fracture calluses of COX-2 knockout mice, showing that shunting can go in both directions [12]. This phenomenon has also been shown in multiple studies involving siRNAs as well as celecoxib, a selective COX-2 inhibitor, indicating shunting is not an experimental artifact [47–49]. While the interplay of AA metabolic pathway components remains to be fully elucidated, it is clear that the relationship between the two arms is quite important. The simultaneous targeting of COX-2 and FLAP by miR-146a in lung cells therefore suggests there is an intricate network of control over AA metabolism and balance of eicosanoid signaling.

In addition to its role in regulation of COX-2 expression in lung cancer cells [19], miR-146a has recently been identified as a negative regulator of immune function, innate and adaptive immune responses, and hematopoiesis [50, 51]. The most well established targets of miR-146a are IRAK1/2 (IL-1 receptor-associated kinase 1/2) and TRAF6 (TNF receptor-associated factor 6) mRNAs, which encode important adaptor molecules in the Toll-like receptor pathway and cytokine responses that lead to NF-κB activation [43, 52–55]. miR-146a itself is an NF-κB-dependent gene, and is part of a negative feedback loop controlling this pathway [43]. Here, we have demonstrated for the first time that miR-146a specifically mediates and downregulates the production of pro-inflammatory LTs. Thus, we postulate that miR-146a acts in both arms of the AA biochemical pathway to fine-tune the response to pro-inflammatory stimuli so as to avoid a chronic and/or overstimulatory response. In lung cancer cells, this regulation is likely affected in that there is significantly reduced miR-146a expression due to promoter hypermethylation, leading to overexpression of the AA metabolic proteins and increased production of their downstream products.

Additionally, both of our studies on miR-146a in lung cancer cells revealed a significant decrease in cellular release of pro-inflammatory PGs and LTs in response to miR-146a treatment. This further supports the biological relevance of our findings. PGE_2_ and LTB_4_ act on epithelial cells in an autocrine and paracrine manner by binding to their specific G protein-coupled receptors, which activate signaling cascades that directly control cell proliferation, survival, migration, and invasion (reviewed in reference [13]). PGE_2_ and LTB_4_ can also participate in crosstalk with the surrounding stromal cells leading to increased angiogenesis [56] and immunosuppression [57] within the tumor microenvironment. Published work from other labs has shown that restoration of miR-146a expression in lung cancer cell lines results in decreased cell migration, proliferation, and growth, as well as increased apoptosis [58, 59], suggesting that this miRNA may be useful as a novel targeted therapeutic. A growing number of researchers are now advocating for dual inhibition of the PG and LT arms of AA metabolism to treat various cancers. Combined knockdown of 5-LO and COX-2 with siRNAs resulted in a more significant decrease in head and neck squamous cell carcinoma cell proliferation *in vitro* than with COX-2 inhibition alone [48]. COX-2/5-LO dual inhibition also resulted in a more robust decrease in colon tumor growth in a nude mouse xenograft model [49]. Our work now demonstrates that miR-146a is an endogenous dual inhibitor of this biochemical pathway.

## MATERIALS AND METHODS

### Mammalian cell culture

Beas2B and A549 cells (ATCC) were grown in Dulbecco's Modified Eagle's Medium (DMEM, Sigma-Aldrich), HeLa cells (ATCC) were grown in Minimal Essential Medium Eagle (Sigma-Aldrich), and H1299 and H1975 cells (ATCC) were grown in Roswell Park Memorial Institute-1640 Medium (Sigma-Aldrich). All media were supplemented with 10% FBS, 4 mM L-glutamine, and 1% Penicillin/Streptomycin, and cells were incubated at 37°C in a 5% CO_2_ incubator. Please note that our strain of Beas2B cells does not cause tumors in nude mice [60].

### Kaplan-Meier survival analysis

The Kaplan-Meier Plotter online tool specific for NSCLC (www.kmplot.com) was used to analyze any potential correlation between overall survival and FLAP expression. This tool was generated using compiled data from multiple cancer patient transcriptomic studies and R statistical software [40]. FLAP expression was designated to be low or high in patients based on their relation to the median value. The KM Plotter tool then generated survival curves, and logrank P values and hazard ratios (HR) with 95% confidence intervals were calculated and plotted in R.

### Western blot analysis

Cells were washed with 1X PBS then lysed with RIPA buffer (50 nM Tris-HCl pH 7.4, 1% NP-40, 0.5% sodium deoxycholate, 0.1% SDS, 150 mM NaCl, 1% protease inhibitor cocktail). Cells were lysed for 30 minutes on ice with occasional stirring. Lysed cells in RIPA were centrifuged at 14,000 x g at 4°C for 15 min. Protein concentration was determined using detergent-compatible (DC) Bradford Assay analysis (Bio-Rad) according to the manufacturer's protocol. Western blot analysis was performed by separating protein samples with 10% SDS-PAGE. Wet electrophoretic transfer to nitrocellulose membrane (80 V, 2 hours, 4°C) was performed, then followed by blocking with 5% non-fat milk + PBSt (5% non-fat dry milk, 1X PBS, 0.1% Tween-20 [Sigma-Aldrich]) for at least 1 hour at room temperature. Primary and secondary antibodies were diluted in 5% non-fat milk + PBSt. Blots were incubated in primary antibodies overnight at 4°C. Post-primary incubation, the blots were washed 3 times for 5 min with PBSt. Blots were then incubated in secondary antibodies for 1-2 hours at room temperature, then washed the same as post-primary incubations. Proteins were detected using HyGlo Quick Spray (Denville Scientific) and a chemiluminescence detection instrument (myECL, Thermo Fisher Scientific). FLAP (EPR5640) rabbit primary monoclonal antibody was purchased from Abcam and diluted 1:1000. α-tubulin horseradish peroxidase (HRP)-conjugated and GAPDH HRP-conjugated antibodies were purchased from ProteinTech and diluted 1:2000. Goat anti-rabbit HRP-conjugated secondary antibody was purchased from Thermo Fisher Scientific and diluted 1:2000. All antibodies were diluted in 5% non-fat milk + PBSt. All Western blots were performed at least three times. Quantification was performed using the gel analysis tool on ImageJ software.

### Quantitative real-time RT-PCR (qRT-PCR)

Total RNA was isolated from cells using TRIzol (Invitrogen) following the manufacturer's protocol. Complementary DNA (cDNA) was synthesized by reverse transcription of RNA using M-MLV Reverse Transcriptase according to the manufacturer's protocol (Invitrogen). For mature miRNA analysis, cDNA was synthesized by reverse transcription of RNA using the miScript kit (Qiagen). qRT-PCR was performed using a Bio-Rad CFX96 Real-Time C1000 Touch Thermal Cycler and the following cycling conditions: (1) 95°C for 15 min, (2) 40 cycles of 94°C for 15 sec, 55°C for 30 sec, 70°C for 30 sec (collection step). Please see Supplementary Table 1 for relevant primer sequences. Expression was measured with SYBR Green/ROX qPCR master mix (Thermo Fisher Scientific). No template and no Reverse Transcriptase (–RT) controls were implemented to ensure samples were not contaminated. Melt curve analysis and electrophoresis of amplified products were performed as well. Quantitative Comparative CT (ΔΔCT) analysis was used to analyze gene expression changes relative to GAPDH or U6. qRT-PCR data represent the average of at least three independent experiments. Each sample was measured with *n* ≥ 2 technical replicates per target gene per independent experiment.

### MicroRNA transfection

Synthetic versions of miR-146a and non-targeting miRNAs were purchased from Dharmacon. Hsa-miR-146a-5p mature microRNA sequence: 5′-UGAGAACUGAAUUCCAUGGGUU-3′. Dharmacon′s miRIDIAN microRNA Mimic Negative Control #1 (sequence is not provided) was used as a non-targeting miRNA. A miRNA inhibitor (antagomiR) specific for miR-146a, anti-hsa-miR-146a-5p, was purchased from Qiagen. Lung cell lines were transiently transfected with synthetic miRs at the indicated concentrations using INTERFERin transfection reagent (Polyplus) according to the manufacturer's protocol. Western blot and qRT-PCR analyses were performed on cells 48 hours post-transfection.

### Doxycycline-induced miR-146a expression

Clones of H1299 cells inducibly expressing miR-146a in the presence of doxycycline were generated using the Retro-X Tet-On 3G inducible expression system (Clontech). The pRetroX Tet 3G vector was used to produce retroviruses according to the manufacturer's suggested protocol. A preliminary cell line stably expressing the Tet transactivator protein (H1299-Tet) was created by transducing H1299 cells with these retroviruses and selecting with 500 μg/mL G418. A 616 bp fragment including the full miR-146a genomic sequence and flanking regions was cloned into the pRetroX TRE 3G vector using the BamHI and EcoRI sites. TRE-empty and TRE-miR-146a retroviruses were produced and used to transduce H1299-Tet cells, followed by selection with 1 μg/mL puromycin. Cells surviving both G418 and puromycin selection were diluted and plated in 96-well plates such that there was approximately one cell per well. Single clones were grown up and analyzed individually. H1299 Tet/TRE-empty and H1299 Tet/TRE-miR-146a cells were cultured, where indicated, in 1 μg/mL doxycycline. All cells were cultured in RPMI supplemented with 10% Tet System Approved FBS (Clontech) and 4 mM L-glutamine.

### Plasmids

The pLightSwitch_3UTR *Renilla* luciferase reporter vector was purchased from SwitchGear Genomics. The FLAP and GAPDH 3′ UTR fragments were cloned into the multiple cloning site downstream of RenSP, the optimized *Renilla* luciferase open reading frame under the control of the RPL10 constitutively active promoter. pLightSwitch_FLAP-146a MUT 3′ UTR, a construct containing the FLAP 3′ UTR fragment with a 4-nt mutation (TTCT – CCGC) in the miR-146a seed sequence, was generated using the GeneArt site-directed mutagenesis system (Invitrogen). Please see Supplementary Table 1 for relevant primer sequences.

### Luciferase assays

HeLa cells were seeded in a 12-well plate format at a density of 0.5 × 10^5^ cells/well. Twenty-four hours after seeding, cells were transfected with a synthetic miRNA (miR-146a, non-targeting miRNA, or anti-miR-146a) at 50 nM as described above (see microRNA transfection). Twenty hours post-miR transfection, cells were transfected with luciferase constructs (pLightSwitch_FLAP-WT 3′ UTR, pLightSwitch_FLAP-146a MUT 3′ UTR, or pLightSwitch_GAPDH 3′ UTR) using LipoD293 transfection reagent (SignaGen Laboratories) according to the manufacturer's suggested protocol. Thirty hours post-DNA transfection, cells were washed with cold 1X PBS and lysed with 250 μL 1X Passive Lysis Buffer (Promega). *Renilla* luciferase activity (luminescence) was measured using the Renilla-Glo luciferase assay system (Promega). Briefly, 50 μL of each lysate was added to a well of a 96-well black-bottom plate and incubated with 50 μL of 1X Renilla-Glo Luciferase Assay Reagent (Promega) for 10 min at room temperature in the dark. Luminescence was then read using a plate reader. *Renilla* luciferase activity from pLightSwitch_FLAP-WT 3′ UTR or pLightSwitch_FLAP-146a MUT 3′ UTR was normalized to activity obtained from samples transfected with pLightSwitch_GAPDH 3′ UTR under the same miR-condition. Samples were further normalized to the protein concentration of each lysate as determined by a Bradford assay. All assays were conducted in independent triplicates, and each individual experiment was repeated at least three times.

### Enzyme-linked immunosorbent assay (ELISA)

Transiently transfected A549 cells were incubated in serum-free media and stimulated with 4 μM calcium ionophore (A23817, Sigma) for 30 min prior to collecting supernatants [61]. H1299 Tet-On cells were incubated in serum-free media containing 10 μM arachidonic acid (Cayman Chemical) for 30 min. Supernatants were removed from the cells and centrifuged at 2,000 x g, 10 min, 4°C. These cell-free supernatants were then analyzed using the Leukotriene B4 EIA Kit (Cayman Chemical) according to the manufacturer's provided protocol. The data represent the average of three independent experiments. Each sample was measured with *n* ≥ 2 technical replicates.

### Methylation analysis

A549 cells were seeded in 60 mm dishes at a density of 3 × 10^5^ cells/dish. The next day, cells were treated with indicated concentrations of 5-aza-2’-deoxycytidine (Sigma-Aldrich). DMSO alone was used as a control treatment. All cells were harvested 24 hours post-treatment and RNA analysis was performed as described above.

Analysis of a 491 bp sequence upstream of the miR-146a transcriptional start site [43] was performed for the presence of CpG sites. Methyl Primer Express v1.1 [62] was used to design methylation-specific PCR (MSP) primers for two regions of this promoter sequence. Please see Supplementary Table 1 for primer sequences. Genomic DNA (gDNA) was isolated from lung cell lines using the DNeasy Blood and Tissue Kit (Qiagen) following the protocol for purification of total DNA from cultured animal cells. These gDNA samples were modified using the EpiTect Bisulfite Kit (Qiagen) following the main protocol provided by the manufacturer. Modified gDNA was amplified with MSP primers using the following cycling conditions: (1) 94°C for 2 min, (2) 35 cycles of 94°C for 30 sec, 55°C for 30 sec, 72°C for 30 sec. PCR products were analyzed with agarose gel electrophoresis and ethidium bromide staining. Band intensities were quantified using the gel analysis tool on ImageJ software. The experiment was repeated three times for each region.

### Data analysis

Data are expressed as mean ± 1SD. Significance was determined using a two-tailed independent sample Student's *t*-test using GraphPad Prism 7.0a. *P*-values < 0.05 were considered significant.

## SUPPLEMENTARY MATERIALS FIGURES AND TABLES


