# Supplementary material for: miR-146a suppresses 5-lipoxygenase activating protein (FLAP) expression and Leukotriene B4 production in lung cancer cells
Source: Oncotarget. 2018 Jun 1;9(42):26751–69. doi: 10.18632/oncotarget.25482 (PMC6003571; doi:10.18632/oncotarget.25482)
Supplement: Supplementary file 1 [file oncotarget-09-26751-s001.pdf]

miR-146a suppresses 5-lipoxygenase activating protein (FLAP) expression and Leukotriene B4 production in lung cancer cells

SUPPLEMENTARY METHODS

Genomic DNA extraction and polymerase chain reaction (PCR)

Genomic DNA was isolated from H1299 Tet/TRE-empty and H1299 Tet/TRE-miR-146a cells using the DNeasy Blood and Tissue Kit (Qiagen) following the protocol for purification of total DNA from cultured animal cells. PCRs were performed using Maxima Hot Start Taq DNA Polymerase (Thermo Fisher) in order to confirm the incorporation of the relevant expression vector sequences in the genome. Please see Supplementary Table 1 for relevant primer sequences. For the Tet and TRE-empty primer sets, the following PCR cycling conditions were used: (1) 95°C for 4 min, (2) 35 cycles of 95°C for 1 min, 55°C for 1 min, 72°C for 30 sec, (3) 72°C for 15 min. For the TRE-146a primer set, the following PCR cycling conditions were used: (1) 95°C for 4 min, (2) 35 cycles of 95°C for 1 min, 65°C for 1 min, 72°C for 1 min, (3) 72°C for 15 min. PCR products were analyzed with agarose gel electrophoresis and ethidium bromide staining.

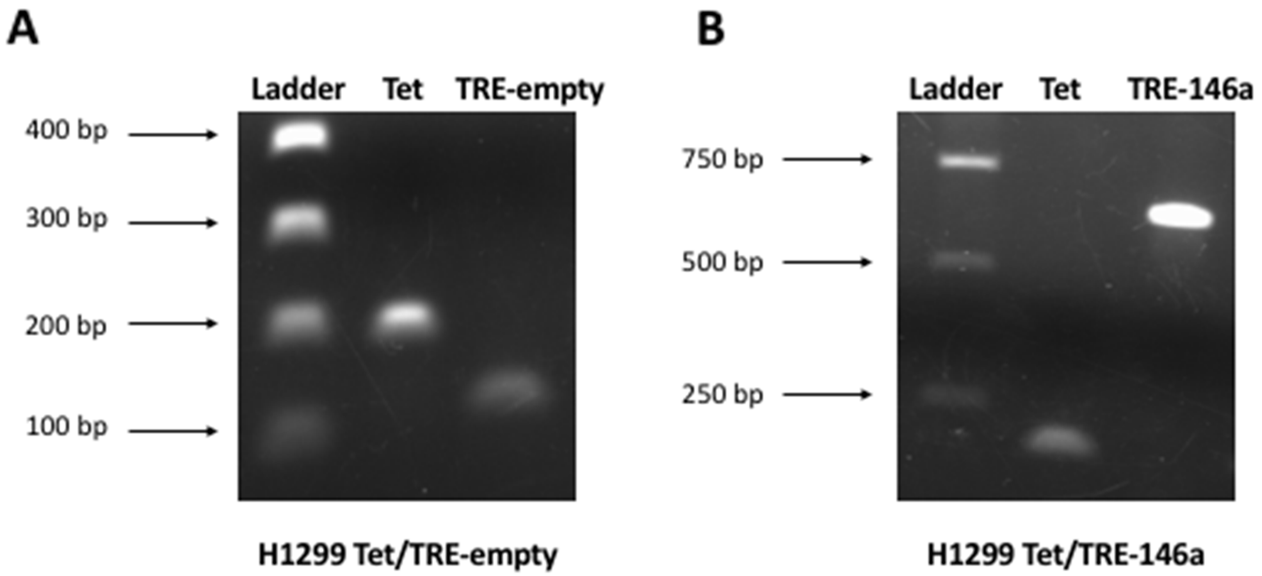

Supplementary Figure 1: H1299 Tet/TRE inducible cell line genomic DNA PCRs. Genomic DNA was extracted from H1299 Tet/TRE-empty and H1299 Tet/TRE-146a stable cell lines. **(A)** H1299 Tet/TRE-empty PCRs for pRetroX Tet 3G and pRetroX TRE 3G empty vector run on 1% agarose gel. **(B)** H1299 Tet/TRE-146a PCRs for pRetroX Tet 3G and pRetroX TRE 3G miR-146a insert sequence (includes restriction enzyme sites) run on 1% agarose gel. Representative reactions shown; three individual clones were tested for each cell line.

Supplementary Table 1: Relevant primer sequences used for various applications

Primer Name	Sequence (5' – 3')	Application	Source
FLAP FWD	AAGTGGAGCACGAAAGCAGGAC	qRT-PCR	Origene
FLAP REV	AGACCAGAGCACAGCGAGGAAA	qRT-PCR	Origene
GAPDH FWD	CCACCCATGGCAAATTCCATGGCA	qRT-PCR	Literature (Young et al. 2012)
GAPDH REV	TCTAGACGGCAGGTCAGGTCCACC	qRT-PCR	Literature (Young et al. 2012)
Mature miR-146a FWD	Proprietary	qRT-PCR	Qiagen Primer Assay
U6 snRNA FWD	Proprietary	qRT-PCR	Qiagen miScript Kit
Universal primer (small RNA REV)	Proprietary	qRT-PCR	Qiagen miScript Kit
FLAP 146A MUT FWD	CTGAATATGGGGTTGGTGCC GCCATCTAATCAATACCTAC	Site-directed mutagenesis	Self-designed
FLAP 146A MUT REV	GTAGGTATTGATTAGATGGC GGCACCAACCCCATATTCAG	Site-directed mutagenesis	Self-designed
Tet FWD	GAAGCCGCTTGGAATAAG	PCR	Self-designed
Tet REV	CAGAGGAACTGCTTCCTTC	PCR	Self-designed
TRE Empty FWD	ATGGAAGTAGCACGTCTCACTA	PCR	Self-designed
TRE Empty REV	AAGGAGCAAAGCTGCTATTG	PCR	Self-designed
TRE 146a FWD	TCTTATACTTGGATCCAGAG ACAAATTCTCCATGTTGC	PCR	Self-designed
TRE 146a REV	CTACCCGGTAGAATTCACAA CAGTACCTGAATCATCGTAA	PCR	Self-designed
146a promoter UM-1 FWD	TTGGGGATTTTTTTGTAGTATGT	MSP	Methyl Primer Express
146a promoter UM-1 REV	AAAAAAACCTAAAAACCCAACACC	MSP	Methyl Primer Express
146a promoter M-1 FWD	GTCGGGGATTTTTTTGTAGTAC	MSP	Methyl Primer Express
146a promoter M-1 REV	AAAAAAACCTAAAAACCCAACG	MSP	Methyl Primer Express
146a promoter UM-2 FWD	TTGGGGGTTTAGATTTTGTATGT	MSP	Methyl Primer Express
146a promoter UM-2 REV	ACCTAACAATATCAACTTCTCTCCAC	MSP	Methyl Primer Express
146a promoter M-2 FWD	TCGGGGGTTTAGATTTTGTAC	MSP	Methyl Primer Express
146a promoter M-2 REV	ACCTAACAATATCAACTTCTCTCCG	MSP	Methyl Primer Express

Abbreviations: FWD: forward; REV: reverse; MSP: methylation-specific PCR; UM: unmethylated; M: methylated.
